# Special Notice

**Published:** 1894-03

**Authors:** Wm. H. Steele

**Affiliations:** Forest City, Iowa


### Special Notice.

As it takes too much time to answer personally all the letters of inquiry I get from brother dentists, I am having a little illustrated leaflet printed explaining Alloy Crowns, Combination Fillings, etc. This will be ready about April 1st, and will be cheerfully mailed to all who address me with stamps.

WM. H. STEELE, Forest City, Iowa.

